# Thermosensitive Injectable Hydrogel for Simultaneous Intraperitoneal Delivery of Doxorubicin and Prevention of Peritoneal Adhesion

**DOI:** 10.3390/ijms19051373

**Published:** 2018-05-04

**Authors:** Chih-Hao Chen, Chang-Yi Kuo, Shih-Hsien Chen, Shih-Hsuan Mao, Chih-Yen Chang, K. T. Shalumon, Jyh-Ping Chen

**Affiliations:** 1 Department of Chemical and Materials Engineering, Chang Gung University, Kwei-San, Taoyuan 33302, Taiwan; chchen5027@gmail.com (C.-H.C.); onesky1997@gmail.com (C.-Y.K.); d9823007@gmail.com (S.-H.C.); reginachangca@gmail.com (C.-Y.C.); shalumon@gmail.com (K.T.S.); 2 Department of Plastic and Reconstructive Surgery and Craniofacial Research Center, Chang Gung Memorial Hospital, Linkou, Chang Gung University School of Medicine, Kwei-San, Taoyuan 33305, Taiwan; raymao0304@gmail.com; 3 Research Center for Food and Cosmetic Safety, Research Center for Chinese Herbal Medicine, College of Human Ecology, Chang Gung University of Science and Technology, Taoyuan 33302, Taiwan; 4 Department of Materials Engineering, Ming Chi University of Technology, Tai-Shan, New Taipei City 24301, Taiwan

**Keywords:** thermosensitive, injectable hydrogel, anti-adhesion, anticancer, chemotherapy, doxorubicin

## Abstract

To improve intraperitoneal chemotherapy and to prevent postsurgical peritoneal adhesion, we aimed to develop a drug delivery strategy for controlled release of a chemotherapeutic drug from the intraperitoneally injected thermosensitive poly(*N*-isopropylacrylamide)-based hydrogel (HACPN), which is also endowed with peritoneal anti-adhesion properties. Anticancer drug doxorubicin (DOX) was loaded into the hydrogel (HACPN-DOX) to investigate the chemotherapeutic and adhesion barrier effects in vivo. A burst release followed by sustained release of DOX from HACPN-DOX was found due to gradual degradation of the hydrogel. Cell culture studies demonstrated the cytotoxicity of released DOX toward CT-26 mouse colon carcinoma cells in vitro. Using peritoneal carcinomatosis animal model in BALB/c mice with intraperitoneally injected CT-26 cells, animals treated with HACPN-DOX revealed the best antitumor efficacy judging from tumor weight and volume, survival rate, and bioluminescence signal intensity when compared with treatment with free DOX at the same drug dosage. HACPN (or HACPN-DOX) also significantly reduced the risk of postoperative peritoneal adhesion, which was generated by sidewall defect-cecum abrasion in tumor-bearing BALB/c mice, from gross and histology analyses. This study could create a paradigm to combine controlled drug release with barrier function in a single drug-loaded injectable hydrogel to enhance the intraperitoneal chemotherapeutic efficacy while simultaneously preventing postsurgical adhesion.

## 1. Introduction

Postoperative adjuvant chemotherapy has been effectively applied for cancer patients with residual tumors after ablation surgeries [1,2,3]. Furthermore, intraperitoneal chemotherapy was reported to be a promising strategy to treat peritoneal carcinomatosis in colorectal cancer to reduce the mortality rate [4,5]. The advantage of intraperitoneal administration of a chemotherapeutic drug may involve minimized systemic toxicity with maximized drug dose delivered to tumor nodules in the peritoneal cavity [6]. However, this administration method poses a challenge to maintain a high ratio of intraperitoneal to plasma drug concentration by demanding more drugs be preserved in the peritoneal cavity than what was absorbed into the systemic circulation [7]. With the short intraperitoneal retention time, frequent or continuous dosing may be required to provide the anticipated benefits of intraperitoneal chemotherapy, which underlines the importance of using a formulation that could control drug release while reducing drug absorption to the blood circulation [8]. Therefore, the efficacy of postoperative peritoneal chemotherapy could be improved by entrapping an anticancer drug within an injectable hydrogel depot within the peritoneal cavity to prolong drug release and maintain drug concentration above the therapeutic window [9]. On the other hand, postoperative peritoneal adhesion is another clinical challenge for patients with peritoneal defects after intraperitoneal chemotherapy [10,11,12]. The severe adhesions may interfere with the exposure of residual tumors to the drug, thus inhibiting the effect of intraperitoneal chemotherapy and possibly leading to therapeutic failure and even mortality [13].

Numerous studies have investigated the pervasive role of thermosensitive hydrogels in biomedical applications, within which poly(*N*-isoropylacrylamide) (PNIPAM) is one of the most widely investigated [14,15]. PNIPAM hydrogels feature a lower critical solution temperature (LCST) around 32 °C [16]. Applying a temperature higher than the LCST causes PNIPAM to precipitate out of a solution as a solid gel. By contrast, a PNIPAM polymer solution changes to liquid form at a temperature lower than the LCST, hence this polymer features reversible phase transition behavior [17]. Previous studies have asserted that a PNIPAM polymer solution can undergo phase transition when experiencing an imbalanced intramolecular force (e.g., ionic strength, hydrophobic force, Van der Walls force, and hydrogen bonding) [18]. At a high temperature, heat weakens the intermolecular hydrogen bonds between water molecules and the hydrophilic groups (C=O and N–H) of PNIPAM but strengthens the intramolecular hydrogen bonds in the PNIPAM molecules, which increase the hydrophobic effect induced by the isopropyl groups of the polymer and cause the PNIPAM molecules to aggregate into a solid gel. At a temperature below LCST, PNIPAM molecules unfold and form random coils, and the hydrogen bonds are re-established between water molecules and the hydrophilic groups of PNIPAM, resulting in a free-flowing polymer solution [19,20,21]. The incorporation of carbohydrate polymers, such as hyaluronic acid, chitosan, or alginate, in PNIPAM could improve the biocompatibility of the thermosensitive hydrogel while retaining its thermo-responsive nature and enhance its mechanical strength [22,23,24,25]. This will allow for delivery of a PNIPAM-based copolymer hydrogel solution during minimally invasive surgery by injecting it through a syringe into the peritoneal cavity at a temperature below the LCST, which could form a rigid hydrogel and be retained within the peritoneal cavity at the physiological temperature [26]. This procedure will offer a much easier, cost-effective, and time-saving surgical procedure than open surgery [27].

Doxorubicin (DOX), or doxorubicin hydrochloride, is a type of anthracycline antibiotic. DOX is a cell cycle nonspecific anticancer drug targeting DNA molecule. Because the molecular size of DOX fits the space between the base pairs in a DNA molecule, the drug can be embedded into a DNA molecule and impede DNA replication, thereby inhibiting the biosynthesis of carcinogenic molecules [28]. Consequently, DOX is prevalently used in cancer treatment. Additionally, previous studies have reported that DOX can react with topoisomerase II in the cell nucleus to form a stable structure [29]. Therefore, this drug can be used to inhibit the reactivity of topoisomerase II during cellular division, leading to DNA breaks and cell death at a drug concentration that is relevant clinically.

Given the thermosensitive injectable characteristics of a hyaluronic acid/chitosan hydrogel (HACPN) based on PNIPAM reported earlier [22], we postulate this hydrogel could be used as a depot for controlled release of DOX by intraperitoneal delivery for cancer therapy. Prompted by numerous reports indicating that an injectable hydrogel similar to HACPN could be also used as a barrier material to prevent postsurgical adhesion in the intraperitoneal cavity [30,31,32,33], we also wish to explore the dual mode of this hydrogel in intraperitoneal chemotherapy. Thus, we intended to use an allograft tumor mouse model in this study to demonstrate that DOX-loaded HACPN hydrogel (HACPN-DOX) could not only improve the efficacy of intraperitoneal chemotherapy but also reduce peritoneal adhesion formation.

## 2. Results

### 2.1. In Vitro Studies

**Figure 1:**
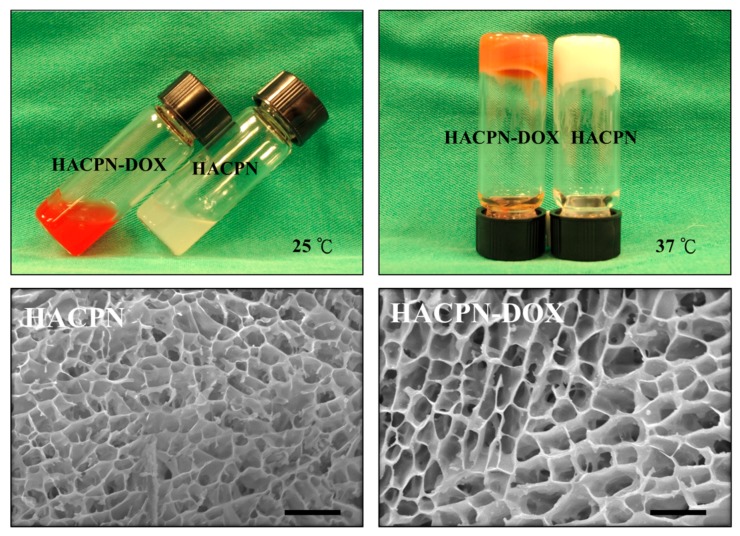
(**Top**) Phase transition behavior from observation of HACPN-DOX (10% (*w*/*v*) HACPN containing 1 mg/mL DOX) and 10% (*w*/*v*) HACPN solutions at 25 and 37 °C; (**Bottom**) Scanning electron micrographs of HACPN and HACPN-DOX hydrogels (bar = 20 μm).

When both 10% (*w*/*v*), HACPN and HACPN-DOX existed as a free-flowing solution at 25 °C (Figure 1). By contrast, both polymer solutions precipitated due to sol-to-gel phase transition when the temperature was raised to 37 °C. It could be observed that the semisolid hydrogels remained stationary when the sample vials were overturned, which implies that HACPN hydrogel has a high enough strength to maintain its structure at the physiological temperature. Consequently, when injected into the peritoneal cavity in the presence of a temperature change to above the LCST, the HACPN-DOX (HACPN) hydrogel is expected to maintain its structure and prevent water from escaping. Prevention of excessive collapse of gel structure above the LCST is important to alleviate drug burst release during gel formation. This could be fulfilled by incorporating chitosan and hyaluronic acid with PNIPAM to form a HACPN copolymer, which was shown to be endowed with abilities to retain more water and resist volume contraction during gel formation [22]. The SEM analysis of the microstructure of the dehydrated hydrogel revealed a highly porous interior structure with interconnected pores below 10 μm in size for HACPN, which remained unchanged when DOX was entrapped into the hydrogel to form HACPN-DOX (Figure 1). The pore size is expected to provide high permeability for nutrient transport while blocking the penetration of fibroblasts that cause postoperative peritoneal adhesion through HACPN hydrogel [33,34].

**Figure 2:**
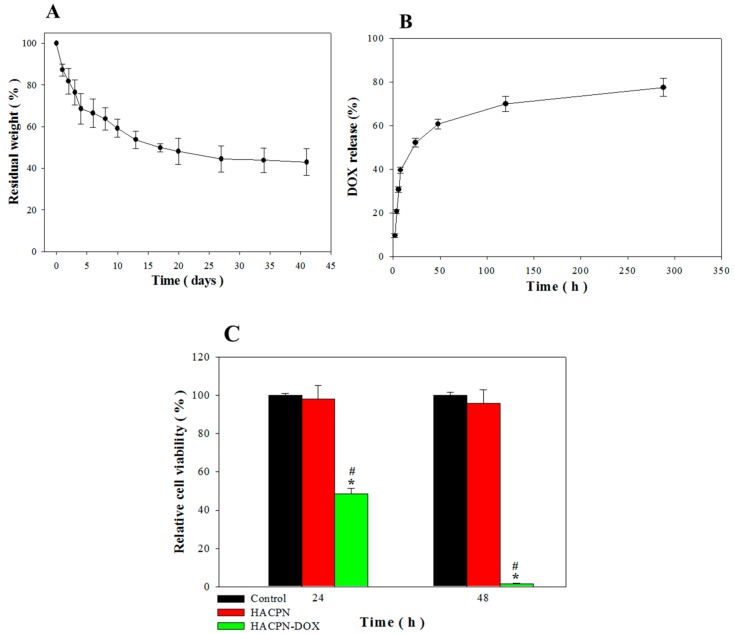
In vitro degradation of HACPN (**A**) and drug release from HACPN-DOX (**B**). (**C**) Cytotoxicity of released DOX toward CT-26 cells determined by MTT assays with cell culture medium (control) taken as 100% relative cell viability. * *p* < 0.01 compared with control, ^#^ *p* < 0.01 compared with HACPN.

The in vitro degradation rate of HACPN was used to examine its effect on drug release and barrier effect. The results in Figure 2A revealed that HACPN retained 70 and 50% of its original weight in phosphate buffered saline (PBS) after 5 and 20 days, respectively. Furthermore, the degradation rates slowed down markedly after day 20 and eventually reached 40% residual weight at day 41, verifying that the copolymer hydrogel could be used for prolonged DOX delivery and anti-adhesion over the entire treatment period. From DOX release from HACPN-DOX in PBS at pH 7.4, a burst release of DOX was observed during the initial period with 40% drug released in 8 h (Figure 2B). Sustained release of DOX was observed thereafter with 80% of the drug released in 12 days. The rapid release initial release of DOX from HACPN hydrogel is expected as a hydrogel with large pores and high water content will provide a pathway for fast diffusion and release of low molecular weight compounds, resulting in rapid DOX release [35]. It is known that DOX is largely a hydrophobic molecule with a hydrophobic anthracycline backbone in addition to several other functional groups including ketone, amine, and hydroxyl groups, which could form electrostatic and hydrogen bond interactions [36]. Therefore, DOX is expected to interact with the hydrophobic regions (from PNIPAM) and the hydrophilic regions (from chitosan and hyaluronic acid) in HACPN. This could contribute to the observed close to linear DOX release rate after 2 days. As the total percentage of DOX released is higher than that of hydrogel degradation, both hydrogel degradation and DOX diffusion contribute to DOX release. Comparing the hydrogel weight loss (Figure 2A) and DOX release percentage (Figure 2B) at the same time point (12 days) suggested that degradation was more important than simple diffusion for DOX release. 

The in vitro cytotoxicity results revealed that the cell viability in the presence of HACPN was not significantly different from the control group (cell culture medium) after 24 and 48 h (*p* > 0.01), indicating that HACPN is not toxic to cells (Figure 2C) [37]. By contrast, the relative cell viability in the HACPN-DOX group was significantly less than those of the control and HACPN groups, which was 48 and 2% that of the control group after 24 and 48 h, implying that the cytotoxicity toward CT-26 was caused by the released DOX but not by HACPN. In order to extend the release time of DOX from HACPN in vivo, covalent binding of DOX to chitosan or hyaluronic components in HACPN could be employed.

### 2.2. Antitumor Effects In Vivo Studies

**Figure 3:**
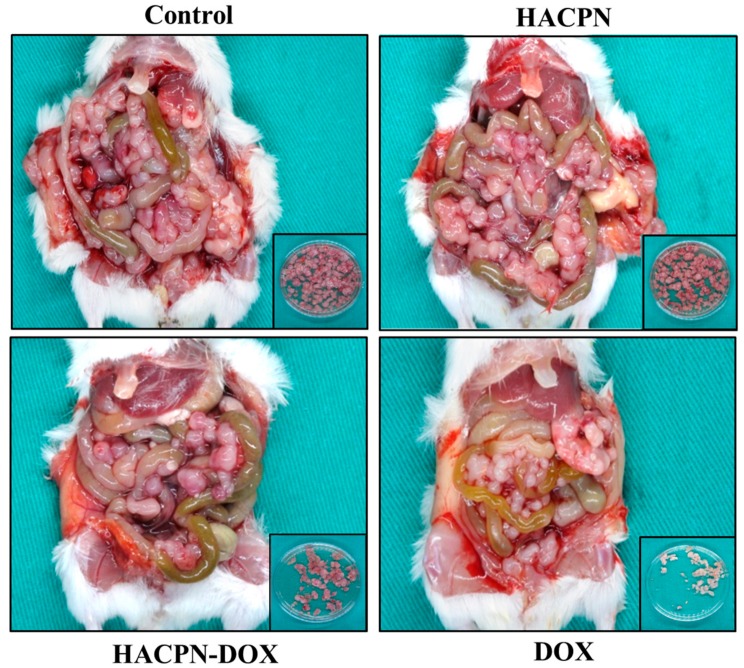
Gross evaluation of the peritoneal tumors removed from peritoneal carcinomatosis mice treated with 200 μL of saline (control), 200 μL of 10% (*w*/*v*) HACPN, 200 μL of 10% (*w*/*v*) HACPN-DOX containing 1 mg/mL DOX, or 200 μL of 1 mg/mL DOX. Insets are the peritoneal tumors removed from the mice in each group.

The peritoneal carcinomatosis model was established in BALB/c by intraperitoneal injection of CT-26 mouse colon carcinoma cells and allograft tumor model was induced for 7 days before treatment [38]. Subsequently, the mice were categorized into four groups according to the received treatment. Fourteen days after treatment, the abdominal cavity was exposed to examine the effects of the treatments. The gross evaluation results revealed that the control and HACPN groups exhibited high amounts of intraperitoneal tumors (Figure 3). In contrast, the HACPN-DOX group exhibited a drastic reduction of tumor amount, indicating DOX released from HACPN hydrogel could exert cytotoxicity on tumors formed from CT-26 in vivo and lead to fewer tumor masses. Compared with the DOX group, which also exhibited fewer amounts of tumors than did the control and HACPN groups, the antitumor effect is more pronounced by using HACPN-DOX than DOX at the same drug dosage. This implies that the dosage of DOX or the duration of the treatment might be insufficient using DOX alone and the drug delivery system using HACPN could provide effective adjuvant chemotherapy treatment for peritoneal carcinomatosis. The appearance and colors of the retrieved tumor masses in the inserts of Figure 3 also revealed a distinct feature. Specifically, the tumors removed from the control and HACPN groups were larger and reddish, whereas those removed from the DOX and HACPN-DOX groups were smaller, with the DOX group showing pink and HACPN showing white color. This implies that DOX not only suppressed tumor growth but also inhibited tumor angiogenesis, as reported in a recent literature [39], and the antitumor effect was consistent from gross observation of the amount and the color of tumors.

The tumor masses were removed from the euthanized mice for determination of the weight and volume of tumors in their abdominal cavities (Table 1). Both tumor weight and volume did not differ significantly between control and HACPN groups. Nonetheless, the tumor weight and volume of the DOX and HACPN-DOX groups were significantly less than either the control or the HACPN group, arising from the antitumor effect of DOX. Most importantly, the tumor weight and volume of the HACPN-DOX group showed a significant decrease from the DOX group, indicating that HACPN-DOX is more effective in suppressing tumor growth [40].

**Figure 4:**
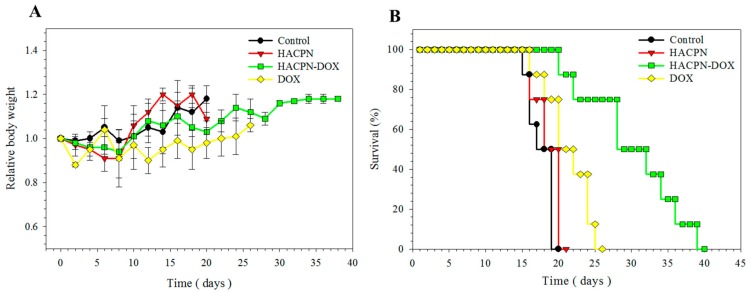
The relative body weight (**A**) and survival rate (**B**) of peritoneal carcinomatosis mice treated with 200 μL of saline (control), 200 μL of 10% (*w*/*v*) HACPN, 200 μL of 10% (*w*/*v*) HACPN-DOX containing 1 mg/mL DOX, or 200 μL of 1 mg/mL DOX.

In addition to investigating tumor growth in mice, we also examined changes in mice body weight. The results revealed that, regardless of the treatment received, the mice did not exhibit a decrease in body weight and exhibited normal body weight increase throughout the treatment period (Figure 4A) [40]. Nonetheless, the weights of mice in the control and HACPN groups markedly increased during the later treatment period, implying that peritoneal tumors might have induced excess ascites formation in those mice. On the contrary, the DOX or HACPN-DOX groups did not exert noticeable weight changes within this period. The normal weight gain of the HACPN-DOX group also endorsed the efficacy and safety of this treatment. 

The mouse survival rates were compared among different treatments in Figure 4B. The time for the first animal’s death was close for the control and HACPN groups, which was at day 15 and day 16. The longest individual survival times of the control and HACPN groups were 20 and 21 days, respectively. The lifespans of the mice in the DOX group were slightly improved, enabling them to survive up to 26 days. By contrast, the mice in the HACPN-DOX group showed the longest individual survival time of up to 40 days. The median survival times were also calculated, which were 18, 19, 29, and 21 days for the control, HACPN, HACPN-DOX, and DOX groups, respectively. Taken together, the results from survival rate assessment indicated that HACPN-DOX can augment the therapeutic effect of DOX by sustained release of DOX and extend the lifespan of tumor-bearing mice to nearly twice as long compared with those of the control and HACPN groups [41].

**Figure 5:**
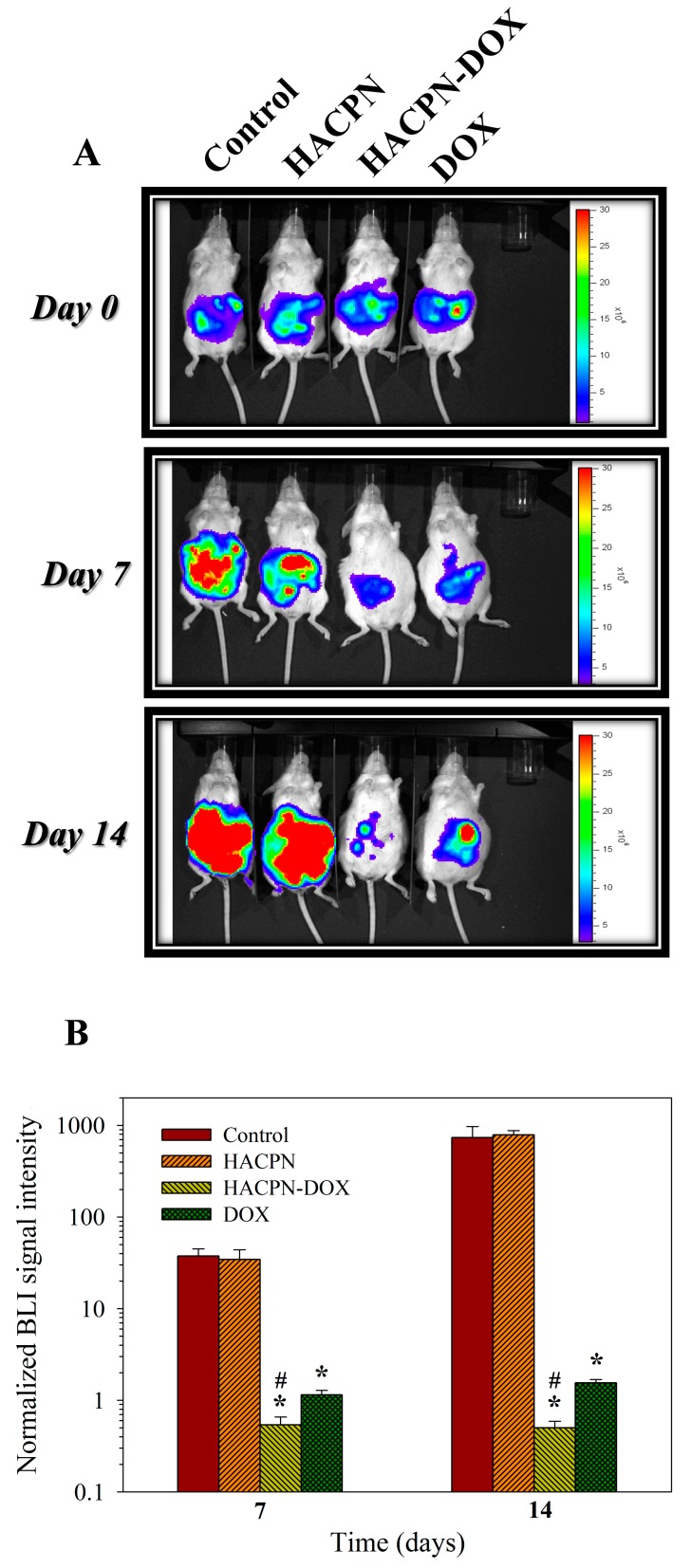
(**A**) Intraperitoneal tumor growth of peritoneal carcinomatosis mice (CT-26/Luc cells) was monitored by bioluminescence imaging (BLI). (**A**) Representative BLI results obtained from mice intraperitoneally injected with 200 μL of saline (control), 200 μL of 10% (*w*/*v*) HACPN, 200 μL of 10% (*w*/*v*) HACPN-DOX containing 1 mg/mL DOX, or 200 μL of 1 mg/mL DOX at day 0, 7, and 14 using in vivo imaging system (IVIS). (**B**) The total bioluminescent signal intensity was normalized by that at day 0 to calculate the normalized BLI signal intensity at day 7 and 14 (mean ± SD, *n* = 8). * *p* < 0.01 compared with control and HACPN, ^#^ *p* < 0.05 compared with DOX.

There is concern over the surgical skills to completely remove the tumor from the peritoneal cavity after sacrificing the animal, which would influence the accuracy of tumor mass and volume measurement. Therefore, we further constructed a CT-26 cell variant (i.e., CT-26/Luc), which was transfected with a luciferase report gene, to facilitate in vivo imaging system (IVIS) imaging and noninvasive monitoring of the treatment efficacy from the tumor bioluminescence imaging (BLI) intensity (Figure 5A). The use of CT-26/Luc for the allograft mouse model has been supported by a previous report, which indicated that transfection with a luciferase gene and bioluminescence did not lead to change in cell growth rate of CT-26/Luc from that of CT-26 [42]. The IVIS images on day 0 displayed similar BLI intensities among all groups, verifying successful induction of peritoneal carcinomatosis in all mice with similar amounts of tumors. In other words, the mice experienced a similar disease condition before receiving the treatment. The luminescence intensities on day 7 confirmed that the tumors in the control and HACPN groups continued to increase and were markedly higher than those in the HACPN-DOX and DOX groups. Thereafter, the HACPN-DOX group showed reduction in tumor size as revealed from diminished luminescence intensity on day 14. By contrast, the luminescence intensity of the DOX group increased from day 7 to day 14, indicating that the therapeutic effect of DOX treatment ceased after 7 days. Not surprisingly, the luminescence intensities of tumors in the control and HACPN groups continuously increased after day 7 in the absence of DOX.

As shown in Figure 5B, the normalized BLI signal intensity increased continuously from day 7 to day 14 for the control and HACPN groups due to active growth of MCF-7/Luc cells (Figure 5B). Treatment by injection of HACPN apparently did not lead to any effect on tumor growth as the normalized BLI signal intensity did not show a significant difference from the control group at both time points (*p* < 0.01). This behavior is expected as there was no DOX for treatment and HACPN showed no cytotoxicity toward CT-26 cells (Figure 2C). For the HACPN-DOX group, the mean BLI signal intensity remarkably decreased to less than that at day 0 (i.e., normalized BLI signal intensity less than 1) 7 days after intraperitoneal administration (Figure 5B). Indeed, we can expect the DOX released from HACPN will exert its cytotoxic effect on CT-26 to result in slower tumor growth and lower BLI intensity. Most importantly, the normalized BLI signal intensity was maintained below 1 from day 7 to day 14 for the HACPN-DOX group, indicating continuous shrinkage of tumor size. For the DOX group, the normalized BLI signal intensity only decreased to less than 1 at day 7, but followed by an increase of BLI intensity to a value higher than its initial value at day 14. This rebound in tumor size implies that intraperitoneal injection of DOX was only effective for a short period of time after drug injection. The difference in tumor growth inhibition between the HACPN-DOX and DOX groups underlines the importance of using HACPN as a depot for sustained release of DOX to enhance tumor treatment efficacy. There is a significant difference in BLI signal intensity between DOX and control, and DOX and HACPN groups at both time points. However, only the HACPN-DOX group showed significant BLI signal intensity difference from the control, HACPN, and DOX groups at all times. From IVIS imaging of tumor mice bearing CT-26/Luc cells, we could confirm the antitumor effect to be consistent with previous results using CT-26 and different methods that assay tumor treatment efficacy (Figure 3 and Figure 4; Table 1).

### 2.3. Anti-Adhesion Effects In Vivo Studies 

**Figure 6:**
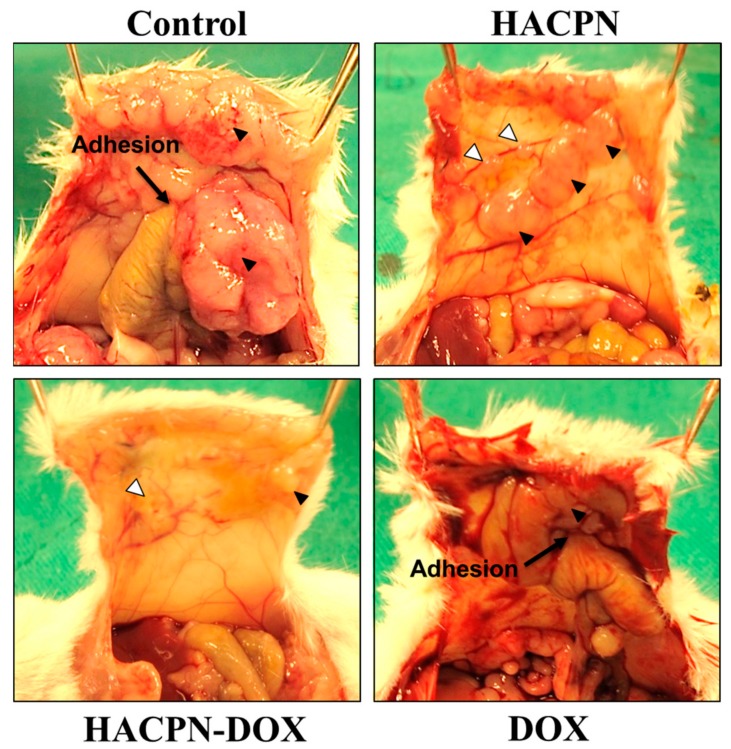
Gross observation of adhesion formation in peritoneal carcinomatosis mice in the saline (control), HACPN, HACPN-DOX, and DOX groups. The black arrows show adhesion formation between peritoneum and cecum. The black arrowheads indicate the peritoneal tumors while white arrowheads indicate the residual hydrogel.

After successful demonstration of the remarkable antitumor effects of HACPN-DOX, we further assess the hydrogel’s ability to prevent peritoneal adhesion in carcinomatosis mice. The tumor-bearing mice, after induction of peritoneal carcinomatosis, were subject to laparotomies with abrasion of cecum and the adjacent parietal peritoneum, followed by the same antitumor treatments as before. The animals were euthanized 2 weeks posttreatment and the extent of postsurgical adhesion was first assessed by gross evaluation (Figure 6). That residual hydrogel in HACPN and HACPN-DOX groups in Figure 6 (white arrowheads) underlines the importance of the slow in vivo degradation rate of hydrogel when acting as a physical barrier for prolonged prevention of peritoneal adhesion, which echoed the in vitro degradation rate of HACPN in Figure 2A.

The grading from gross observation indicated the adhesion score of all treatment groups was significantly lower than that in the control group (*p* < 0.001, Mann–Whitney U test) (Table 2). Although most mice in the control (87.5%) and DOX (62.5%) groups developed adhesion with a score of 3, none in groups treated with the hydrogel (HACPN and HACPN-DOX groups) developed such adhesion (*p* < 0.001, Fisher’s exact test). Five and six mice (62.5 and 75%) in the HACPN and HACPN-DOX groups, respectively, showed no tissue adhesion at all (score = 0), compared with 0% in the control and DOX groups (*p* < 0.001, Fisher’s exact test). For two mice that developed minor adhesions (score 1) in each of the HACPN and HACPN-DOX groups, three were between nonabraded cecum and small intestine and one was between the abdominal wall defect margin and omentum. One mouse in the HACPN group developed mild adhesion (score 2) between the tumor on the peritoneum and the cecum but none in the HACPN-DOX group. All locations where score 1 and score 2 adhesions developed from gross evaluation were confirmed to be sites that were not covered by the hydrogel. Overall, we could conclude that both HACPN and HACPN-DOX treatments could take advantage of the anti-adhesion barrier effect of HACPN when the polymer solution showed sol-to-gel phase transition and adhered to the injured surface, which effectively covered the injured surface throughout the peritoneal healing period [37].

**Figure 7:**
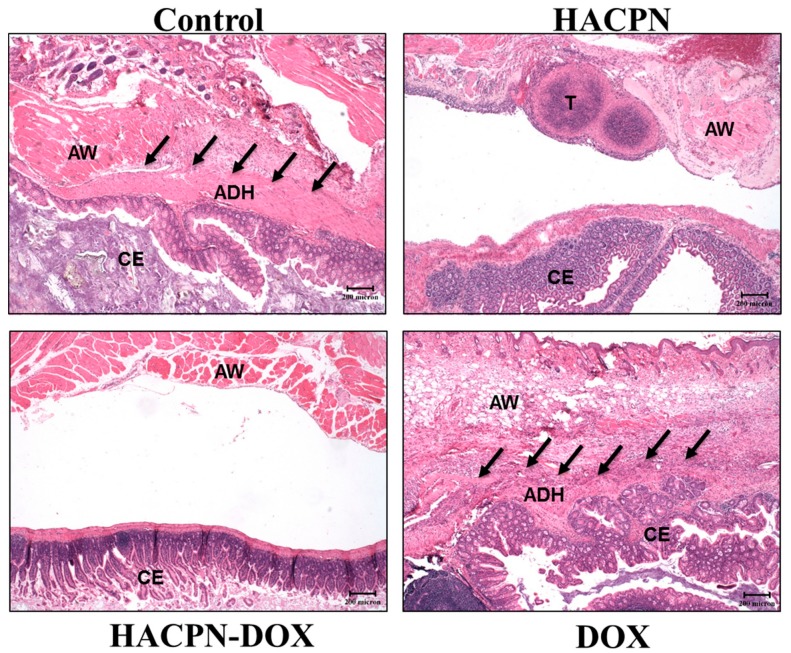
The hematoxylin and eosin (H&E) staining of tissue sections from peritoneal carcinomatosis mice after saline (control), HACPN, HACPN-DOX, and DOX treatments. Scale bar = 200 μm. Black arrows indicate the adhesion between peritoneum and cecum. ADH = adhesion; AW = abdominal wall; CE = cecum; T = tumor.

From histological examination of tissue sections retrieved from the injured sites, samples in the hydrogel groups (i.e., HACPN and HACPN-DOX) showed an integral neomesothelial cell layer with no sign of adhesion, indicating the proper healing of peritoneal wounds (Figure 7). However, samples harvested from the peritoneal adhesion areas in the control and DOX groups showed fibrous tissue of different thickness with penetration of adhesion into the abdominal wall muscle (Figure 7). The adhesion scores from histology also underline the consistency of hydrogels in preventing peritoneal adhesion formation with a significantly lower median score for the HACPN and HACON-DOX groups (median score = 0) compared with the other two groups (median score = 3) (Table 2). Overall, HACPN is expected to be a good physical barrier to prevent adjacent fibroblasts from penetrating through its microsized pores and approaching the damaged tissue to result in adhesion formation [43]. HACPN-DOX apparently retained this beneficial function with similar pore size (Figure 1) to exhibit similar anti-adhesion efficacy from gross evaluation and histological analysis (Table 2). Indeed, a thermosensitive injectable hydrogel that shows sol-to-gel phase transition around the physiological temperature facilitates its use when injected through a syringe needle into the peritoneal cavity. Nonetheless, pure PNIPAM hydrogel showed high volume shrinkage during gel formation, which will hinder its application as an anti-adhesion barrier in addition to vast loss of entrapped drug. In contrast, the introduction of chitosan and hyaluronic acid into the polymer backbone of PNIPAM hydrogel (HACPN) not only preserved the thermo-responsiveness with comparable LCST, but also enhanced water retention and reduced volume shrinkage of the formed gel [43]. This endowed the hydrogel with the ability to completely cover the peritoneal tissue and isolate it from surrounding tissues concomitant with delivery of a chemotherapeutic drug such as DOX. Using the peritoneal carcinomatosis animal model, we demonstrate HACPN-DOX treatment could provide remarkable antitumor efficacy in addition to superior anti-adhesion outcomes.

## 3. Materials and Methods

### 3.1. Preparation of and Characterization of HACPN and HACPN-DOX

The synthesis and characterization of HACPN hydrogel followed the procedure described in our previous work [22]. We first synthesized PNIPAM with carboxylic-acid-ended groups by free radical polymerization between *N*-isopropylacrylamide and mercaptoacetic acid in benzene with 2,2′-azobis(2-methylpropionitrile) as an initiator. To synthesize chitosan-grafted PNIPAM (CPN), 2 g PNIPAM with carboxylic-acid-ended groups and 0.2 g chitosan (average molecular weight = 1 × 10^5^ Da) were dissolved in 20 mL 2-(*N*-morpholino)ethanesulfonic acid (MES) buffer (0.1 M, pH 5) containing 0.55 g *N*-hydroxysuccinimide (NHS) and 0.183 g 1-ethyl-3-(3-dimethylaminopropyl) carbodiimide hydrochloride (EDC). The reaction was carried out at 25 °C and 180 rpm for 12 h, after which CPN was purified by adding NaCl to a final salt concentration of 0.6 M and incubating at 50 °C for 30 min to induce precipitation. The cloudy solution was centrifuged at 16,000× *g* for 20 min, followed by solubilizing the precipitate in 20 mL MES buffer (0.1 M, pH 5) and further purified three times following the same procedure. The CPN prepared above was mixed with 0.1 hyaluronic acid (HA, average molecular weight = 1.3 × 10^6^ Da), 0.55 g NHS, and 0.183 g EDC in 40 mL of 0.1 M MES buffer (pH 5) at 25 °C and 180 rpm for 12 h to synthesize HACPN. Residual HA in the solution was removed by thermal precipitation at 50 °C and centrifugation as before. After dialysis against distilled deionized water for 4 days, purified HACPN was obtained and lyophilized for storage in a desiccator. To prepare HACPN with entrapped DOX (HACPN-DOX), DOX solution was first prepared in phosphate buffer solution (PBS) at 1 mg/mL, followed by dissolving 0.1 g of HACPN powder in the DOX solution to obtain a 10% (*w*/*v*) HACPN–DOX polymer solution [44].

To visualize the microstructure of the hydrogels, a 10% (*w*/*v*) HACPN polymer solution prepared at 37 °C was frozen instantaneously in liquid nitrogen and observed by scanning electron microscopy (SEM). To preserve the original pore structure, the frozen hydrogel was lyophilized in a freeze dryer after removing from the liquid nitrogen, fractured, and coated with gold to 6 to 9 nm thickness. The cross-section morphology of the dried hydrogel sample was examined with a scanning electron microscope (ISM 5410, JEOL, Tokyo, Japan). 

### 3.2. In Vitro Degradation and Drug Release Profile of Hydrogel

A 10% (*w*/*v*) HACPN solution prepared in PBS (200 μL) was placed in a preweighed cell culture insert (Millicell^®^, Merck Ltd., Taiwan, Taipei, Taiwan). The insert was incubated at 37 °C to convert the loaded polymer solutions into gels. Subsequently, 5 mL of 37 °C PBS was added to the insert and the cell inset was placed in a 37 °C incubator and shaken at 50 rpm. The supernatant was removed from the insert at different time points and hydrogels plus the inserts were rapidly frozen and lyophilized. To determine the degradation of HACPN, the residual weight of the hydrogel (W*_t_*) was determined at different time points and the residual weight (%) was calculated from (W*_t_*/W*_i_*) × 100%, where W*_i_*is the initial weight of HACPN. To determine drug release in vitro, 200 μL of 10% (*w*/*v*) HACPN-DOX polymer solution was placed in a cell culture insert (Millicell^®^) for gel formation at 37 °C. The insert was placed in a 6-well cell culture dish and each well was filled with 10 mL of PBS (pH = 7.4). The dish was sealed and shaken at 50 rpm in a 37 °C incubator. One milliliter of the supernatant was removed from each well in the dish at predetermined times and the absorbance of the solution at 490 nm was determined using an Ultraviolet/visible (UV/VIS) spectrophotometer (Evolution 300, Thermo Fisher Scientific, Waltham, MA, USA). The absorbance was converted to DOX concentration using a calibration curve constructed beforehand. After completely removing the remaining solution in the dish, 10 mL of PBS (pH = 7.4) was added to each well and the culture dish was continuously incubated at 37 °C and 50 rpm for drug release. The release of DOX (%) was reported as (W*_t_*/W*_i_*) × 100% with W*_t_* representing the cumulative DOX released weight and W*_i_*representing the initial DOX weight in HACPN-DOX.

### 3.3. In Vitro Cell Culture and Cytotoxicity 

CT-26 mouse colon carcinoma cells (Food Industry Research and Development Institute, Hsinchu, Taiwan) were maintained at 37 °C in RPMI-1640 medium (Gibco, Thermo Fisher Scientific, Waltham, MA, USA) supplemented with 10% fetal bovine serum (FBS, Hyclone, Thermo Fisher Scientific, Waltham, MA, USA), 1% l-glutamine, streptomycin (100 ng/mL), and penicillin (100 unit/mL) in a humidified CO_2_ incubator. The cytotoxicity of HACPN and HACPN-DOX was determined with CT-26 cells cultured in 2 mL of RPMI-1640 medium supplemented with 10% FBS in a 24-well culture plate (1 × 10^4^ cells/well). After overnight incubation in a humidified CO_2_ incubator, Millicell^®^ cell culture inserts filled with 500 μL of HACPN or HACPN-DOX solution were fitted in each well of the 24-well cell culture plate. After 24 and 48 h culture in a CO_2_ incubator, the relative cell viability was determined by 3-(4,5-dimethylthiazol-2-yl)-2,5-diphenyltetrazolium bromide (MTT) assays. Two hundred microliter of 1 mg/mL MTT reagent (Sigma-Aldrich, St. Louis, MO, USA) prepared in cell culture medium was added to each well and the cell culture plate was incubated for an additional 3 h at 37 °C in a CO_2_ incubator. Two hundred microliter of dimethylsulfoxide was added to each well to dissolve the formed blue formazan crystal and the solution absorbance was measured using a microplate reader (Synergy HT, BioTek Instruments Inc., Winooski, VT, USA) at 540 nm. The control was cell culture medium and all procedures were carried out in the dark.

### 3.4. Peritoneal Carcinomatosis Model and Treatment Protocol

The Institutional Animal Care and Use Committee of Chang Gung University approved all animal experiment protocols (IACUC Approval No.: CGU14-078, 09/22/2014). A peritoneal carcinomatosis model was established in 16~18 g female BALB/c mice (BioLASCO Taiwan Co., Ltd., Taipei, Taiwan) by injecting 200 μL of CT-26 cell suspension (2 × 10^5^ cells) intraperitoneally into the lower abdominal cavity of mice. The injected tumor cells were allowed to spread in the abdominal cavity for 7 days before treatment. Mice with peritoneal carcinomatosis were randomly divided into 4 groups with 8 animals in each group. The mouse was subject to intraperitoneal injection of 200 μL of saline (control group); 200 μL of 10% (*w*/*v*) HACPN (HACPN group); 200 μL of 10% (*w*/*v*) HACPN-DOX containing 1 mg/mL DOX (HACPN-DOX group); 200 μL of 1 mg/mL DOX (DOX group). The mice were sacrificed 2 weeks after treatment and all tumors were removed from the peritoneal cavity by excision. The abdominal tumor mass was weighed and the tumor volume was determined by the Archimedes (water displacement) procedure. The body weight and survival time of the mice were also continuously monitored for further study of the antitumor effect. 

### 3.5. Bioluminescence Imaging (BLI) Using the In Vivo Image System (IVIS)

To facilitate noninvasive IVIS imaging, we first constructed a CT-26/Luc cell line with stable neomycin-resistant and firefly luciferase genes using the pGL4.51[luc2/CMV/Neo] plasmid vector (Promega, Madison, WI, USA) and transfection liposome vehicle (E2431, Promega, Madison, WI, USA) through standard protocols [45]. To select transfected cells, 1 mg/mL G418 ((Sigma-Aldrich, St. Louis, MO, USA) selection antibiotic was used for two weeks to isolate the resistant colonies that were tested positive for luciferase activity. Animals were grouped as described before for CT-26 cells after injecting the same number of CT-26/Luc cells intraperitoneally into the mice (*n* = 8 for each group). The bioluminescence imaging (BLI) was carried out noninvasively at day 0, 7, and 14 after treatment. After anesthetizing the mice with inhalational anesthesia, 200 μL of D-luciferin was injected intraperitoneally into the mice (150 mg/kg) and images were acquired noninvasively with a Xenogen IVS-200 IVIS (Caliper Life Sciences, Waltham, MA, USA). The total peak bioluminescent signal intensities in the abdominal region were determined using the Living Image^®^ 4.0 software (PerkinElmer, Waltham, MA, USA) at regions of interest (ROIs) as BLI signal intensities at day 0 (i.e., before treatment), day 7, or day 14 after treatment. To normalize the BLI signal intensity, the BLI signal intensity at day 7 or day 14 was divided by the BLI signal intensity at day 0.

### 3.6. The Anti-Adhesion Effects from Sidewall Defect-Cecum Abrasion Model 

The anti-adhesion effects were evaluated by using a sidewall defect-bowel abrasion model in mice [46]. All experimental protocols followed the regulations of the Institutional Animal Care and Use Committee of Chang Gung University (IACUC Approval No.: CGU12-109, 02/06/2013). The peritoneal carcinomatosis animal model in BALB/c female mice was established as before. One week after CT-26 cells injection, peritoneal adhesion was induced with a 1 × 1 cm^2^ defect on the left lateral abdominal wall of mice by abrading the cecal haustra to spot bleeding. Mice were randomly divided into 4 groups with 8 animals in each group and subject to intraperitoneal injection of 200 μL of saline (control group); 200 μL of 10% (*w*/*v*) HACPN (HACPN group); 200 μL of 10% (*w*/*v*) HACPN-DOX containing 1 mg/mL DOX (HACPN-DOX group); 200 μL of 1 mg/mL DOX (DOX group). The peritoneum and abdominal wall were finally closed using 5-0 Vicryl sutures, followed by closing the skin with 4-0 Ethylon sutures. Two weeks after operation, mice were sacrificed for gross evaluation of the extent of postoperative peritoneal adhesion. The adhesions from gross evaluation were graded by two surgeons in a blinded manner with scores from 0 to 3 based on adhesion severity. No adhesions was graded as 0, gentle blunt dissection needed for freeing adhesions was graded as 1; aggressive blunt dissection needed for freeing adhesions was graded as 2; sharp dissection needed for freeing adhesions was graded as 3 [47]. For histopathological examination, harvested tissues including abdominal defect, cecum defect, and adhesion tissues in between were fixed in 4% paraformaldehyde, followed by embedding in paraffin. The specimens were sectioned and stained with hematoxylin and eosin (H&E). All slides were reviewed by light microscopy and graded by two pathologists in a blinded manner with the adhesion scores from 0 to 3 based on histology examination. No adhesion was graded 0, minimal and loose adhesion was graded 1, moderate adhesion was graded 2, and dense adhesion was graded 3. 

### 3.7. Statistical Analysis

All values were expressed as mean ± standard deviation (SD) from at least five independent experiments. The Student’s *t*-test was used to compare the values between two groups. As adhesion scores seldom follow normal distribution, we made statistical inferences of adhesion scores based on Fisher’s exact test or Mann–Whitney U-tests using SPSS 10.0 software (SPSS Inc., Chicago, IL, USA). Significant differences were statistically announced when the *p* value was less than 0.05.

## 4. Conclusions

We concluded that HACPN-DOX is an ideal injectable drug formulation for intraperitoneal chemotherapy and prevention of postsurgical peritoneal adhesion. The drug-loaded thermosensitive hydrogel showed promising cytotoxicity against CT-26 mouse colon carcinoma cells through controlled release of DOX from the gelled depot and the hydrogel could be gradually degraded in vitro. Using the peritoneal carcinomatosis model in BALB/c mice, we demonstrated the remarkable antitumor efficacy of HACPN-DOX in vivo based on gross evaluation, tumor weight and volume, survival rate, and tumor BLI signal intensity from IVIS imaging. The sidewall defect-cecum abrasion animal model also indicated that the hydrogel showed barrier effect through unrestricted coverage of the injured surface until healing to prevent peritoneal adhesion, which could be confirmed from gross and histology grading analyses. As HACPN shows thermosensitive injectable characteristics, we can use it for noninvasive intraperitoneal delivery. Judging from the improved antitumor and anti-adhesion efficacy, HACPN was confirmed to play a dual role in intraperitoneal drug delivery and anti-adhesion for good prognosis after intraperitoneal chemotherapy of abdominal and pelvic carcinomatosis.

## Figures and Tables

**Table 1:** The weights and volumes of the peritoneal tumors removed from peritoneal carcinomatosis mice ^1^.

Treatment	Tumor Weight (g)	Tumor Volume (cm^3^)
Control	2.50 ± 0.12	2.61 ± 0.16
HACPN	2.60 ± 0.08	2.70 ± 0.10
HACPN-DOX	0.30 ± 0.03 *^,#^	0.46 ± 0.08 *^,#^
DOX	1.13 ± 0.09 *	1.46 ± 0.12 *

^1^ The mouse was injected intraperitoneally with 200 μL of saline (control), 200 μL of 10% (*w*/*v*) HACPN, 200 μL of 10% (*w*/*v*) HACPN-DOX containing 1 mg/mL DOX, or 200 μL of 1 mg/mL DOX. * *p* < 0.05 compared with compared with control and HACPN, ^#^ *p* < 0.05 compared with DOX.

**Table 2:** The number of carcinomatosis mice with different peritoneal adhesion scores for each treatment group from gross view and histology analyses (*n* = 8 each group).

Gross View ^1.^	Control	HACPN	HACPN-DOX	DOX
Score 0	0	5 (62.5%)	6 (75.0%)	0
Score 1	0	2 (25.0%)	2 (25.0%)	0
Score 2	1 (12.5%)	1 (12.5%)	0	3 (37.5%)
Score 3	7 (87.5%)	0	0	5 (62.5%)
**Histology ^2.^**	**Control**	**HACPN**	**HACPN-DOX**	**DOX**
Score 0	0	6 (75.0%)	7 (87.5%)	0
Score 1	0	2 (25.0%)	1 (12.5%)	1 (12.5%)
Score 2	1 (12.5%)	0	0	2 (25.0%)
Score 3	7 (87.5%)	0	0	5 (62.5%)

^1^ The adhesion scores from gross view were graded as 0 with no adhesion, 1 when gentle blunt dissection was required to free adhesion, 2 when aggressive blunt dissection was required to free adhesion, and 3 when sharp dissection was required to free adhesion. ^2^ The adhesion scores from histology were graded as 0 when no adhesion occurred, 1 when minimal and loose adhesion occurred, 2 when moderate adhesion occurred, and 3 when dense adhesion occurred.

## Abbreviations

HA	Hyaluronic acid
PNIPAM	Poly(*N*-isoropylacrylamide)
HA-CPN	Hyaluronic acid-g-chitosan-g-PNIPAM
LCST	Lower critical solution temperature
PBS	Phosphate buffered saline
DOX	Doxorubicin
FBS	Fetal bovine serum
IVIS	In vivo imaging system
BLI	Bioluminescence imaging
UV/VIS	Ultraviolet/visible
H&E	Hematoxylin and eosin
ROI	Region of interest
SEM	Scanning electron microscopy
